# Target delivery of small interfering RNAs with vitamin E-coupled nanoparticles for treating hepatitis C

**DOI:** 10.1038/srep24867

**Published:** 2016-04-26

**Authors:** Liang Duan, Yan Yan, Jingyi Liu, Bo Wang, Pu Li, Qin Hu, Weixian Chen

**Affiliations:** 1 Department of Laboratory Medicine, the Second Affiliated Hospital of Chongqing Medical University, Chongqing, 400010, China

## Abstract

RNA interference (RNAi) represents a promising strategy for the treatment of HCV infection. However, the development of an effective system for *in vivo* delivery of small interfering RNA (siRNA) to target organ remains a formidable challenge. Here, we develop a unique nanoparticle platform (VE-DC) composed of α-tocopherol (vitamin E) and cholesterol-based cationic liposomes (DOTAP-Chol) for systemic delivery of siRNAs to the liver. A HCV-replicable cell line, Huh7.5.1-HCV, and a transient HCV core expressing cell line, Huh7.5.1-Core, were constructed and used to assess the *in vitro* anti-HCV activity of VE-DC/siRNAs. A transient *in vivo* HCV model was also constructed by hydrodynamic injection of pCDNA3.1(+)-3FLAG-Core (pCore-3FLAG) plasmid expressing core protein or pGL3-5′UTR-luciferase (pGL3-5′UTR-luc) plasmid expressing luciferase driven by HCV 5′UTR. Nanoscale VE-DC/siRNA was intravenously injected to assess the liver-targeting property as well as antiviral activity. The nanoscale VE-DC effectively exerted an anti-HCV activity in the *in vitro* cell models. Post-administration of VE-DC/siRNAs also effectively delivered siRNAs to the liver, suppressing core protein production and firefly luciferase activity, without inducing an innate immunity response or off-target and toxicity effects. The VE-DC platform has high potential as a vehicle for delivery of siRNAs to the liver for gene therapy for targeting hepatitis C.

Hepatitis C virus (HCV) is a major cause of chronic liver disease and affects over 185 million individuals worldwide^1^. Persistent HCV infection is a leading cause of chronic hepatitis, liver cirrhosis, and even hepatocellular carcinoma^2^,^3^. Although HCV infections can be partially combatted by the new treatment regimens with direct-acting antiviral drugs (DAAs), the high specificity of DAAs against their viral targets might result in emergence of antiviral resistance, and even these regimens are costly which might also lead to a restricting societal benefit^4^. Thus, developing a new therapy for HCV is a major public health objective.

HCV has a positive strand genome comprising 9.6 kb RNA which is processed by cellular and viral proteases into 10 viral proteins in the order of NH2-Core-E1-E2-p7-NS2-NS3-NS4A-NS4B-NS5A-NS5B-COOH. HCV core is involved in a whole array of host cell functions, including cell apoptosis, cell transformation, dysregulation of lipid metabolism, modulation of immunological function and regulation of cell signaling^5^. NS2-NS5B are putative nonstructural proteins involved in the replication of HCV^6^. Although specific pathogenesis of HCV disease remains unknown, direct interaction of specific viral proteins with host cell system has proven to be accounted for some of its pathophysiological profile of HCV patients^7^,^8^.

Gene therapy involves the use of nucleic acids (DNA or RNA) for the treatment, cure or prevention of human disorders. Depending on the type of disease, this can be achieved either by delivery of a functional or therapeutic gene using various sophisticated tools including naked oligonucleotides, viral and non-viral vectors. Highly efficient gene targeting strategies and site-directed gene editing technologies have been developed and applied clinically. With more than 1900 clinical trials to date, gene therapy has moved from a vision to clinical reality^9^. RNA interference (RNAi) technology is widely used as a tool for gene function analysis, as it can exert a degradative effect on target gene mRNA with exquisite sequence specificity without activating an interferon response. It represents an exciting new technology with therapeutic applications for the treatment of many diseases, including viral infections and cancer^10^,^11^,^12^,^13^. HCV, as a single plus RNA virus that replicates in the cytoplasm of liver cells, is sensitive to RNAi and might be an ideal target for this therapy. Destruction of HCV RNA could induce failure of HCV replication, indicating that the use of siRNA could be a novel regulatory approach for molecular therapeutics^14^,^15^,^16^,^17^. HCV is also prone to mutation and escape from single antivirals, so it also applies to siRNAs and it is generally accepted that combinatorial approaches, or targeting of host dependency factors, are required to prevent emergence of escape mutants. miR-122 represents an essential hepatocyte-specific host factor that is required by HCV replication and represents a target for antiviral therapy^18^. Trials are in progress that demonstrate good efficacy of oligonucleotides that inhibit function of miR-122^19^. Therefore, RNAi-based gene therapy may prove a high potential for treatment of HCV. A key challenge in realizing the full potential of RNAi therapeutics is the efficient delivery of siRNA, the molecules that mediate RNAi. However, siRNA molecules injected in to the blood can be quickly degraded by RNase activity in the serum environment and rapidly excreted in the urine. Consequently, siRNA requires a safe and efficient delivery system for transfer to the target tissues. This remains an obstacle to achieving *in vivo* gene silencing by RNAi-based gene therapy.

Gene delivery vectors are classified into viral and non-viral categories. A viral vector is an efficient approach for gene delivery, but its application may result in severe side effects, including biological safety issues and immunogenicity^20^. Non-viral vectors, such as cationic liposomes, represent an attractive means for gene delivery due to their excellent biocompatibility, low immunogenicity and toxicity, and large carrying capacity for nucleic acids^21^,^22^. Neutral lipids, including dioleoylphosphatidylethanolamine (DOPE), dioleoylphosphatidylcholine (DOPC), and cholesterol, are often used as the components for cationic liposome formulations, where they play an assistant role^23^,^24^. Notably, cholesterol-based cationic liposomes have been used as the major hydrophobic domain of liposomes for gene delivery due to their less toxic effects than other cationic liposomes^24^. The physical properties of cationic liposomes are well understood, so they hold the best promise for clinical application.

The most effective *in vivo* vector for gene delivery for use in HCV therapy would be one that specifically targeted the liver. One candidate is α-tocopherol (Vitamin E, VE), which is an abundant lipid-soluble antioxidant agent that has many physiological pathways from serum to liver^25^,^26^. Here, we designed and synthesized a novel siRNA-packing, nanoscale, cholesterol-based cationic liposome (DOTAP-Chol, DC) formulation for destruction of HCV RNA, and coupled it with VE (VE-DC) promoted delivery of siRNAs to the liver. Importantly, siRNAs delivered by our nanoscale VE-DCs were largely transfected into hepatocytes, both *in vitro* and *in vivo*, where they efficiently silenced the expression of a target HCV gene and potently suppressed HCV replication. The system also exhibited high delivery efficiency, low injection dose requirements, and low toxicity, suggesting that delivery of siRNA by nanoscale VE-coupled cholesterol-based cationic liposomes represents a promising strategy for the clinical development of novel anti-HCV therapeutics.

## Results

### Inhibition of HCV gene expression by efficient siRNA *in vitro*

**Figure 1:**
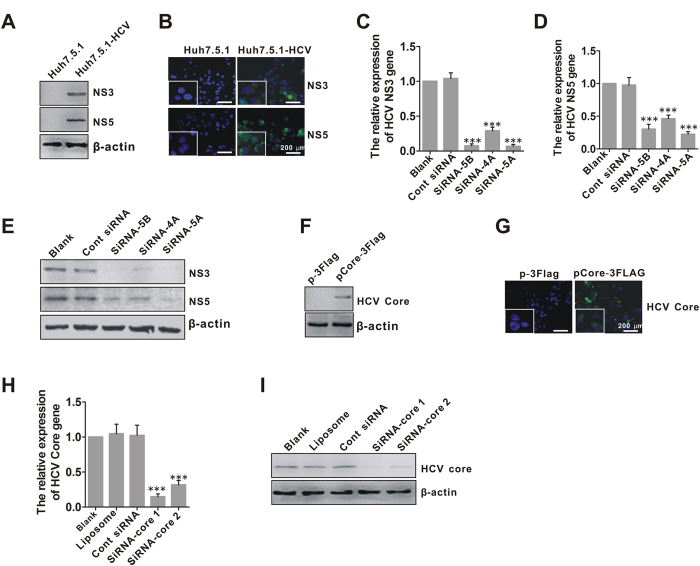
Silencing effects of HCV replicon-specific siRNA on HCV replication in Huh7.5.1-HCV cells and of core-specific siRNA on core expression in Huh7.5.1-Core cells *in vitro*. (**A**) Western blot analysis for NS3 and NS5A protein expression in Huh7.5.1-HCV cells. β-actin was used as an input control. (**B**) immunofluorescent staining for NS3 and NS5A protein expression in Huh7.5.1-HCV cells. The cells were stained with the antibody against NS3 or NS5A, and then FITC-labeled secondary antibody was applied (green fluorescence). The nuclei were counterstained with DAPI (blue). White scale bars = 200 μm. (**C**) Inhibition of NS3 gene expression in Huh7.5.1-HCV cells by replicon-specific siRNAs (siNS5B, siNS4A and siNS5A), determined by real-time PCR. ***p < 0.001, all siRNAs vs.Blank. (**D**) Inhibition of NS5A gene expression in Huh7.5.1-HCV cells by siNS5B, siNS4A and siNS5A, determined by real-time PCR. ***p < 0.001, all siRNAs vs.Blank. (**E**) Inhibition of NS3 and NS5A protein expression in Huh7.5.1-HCV cells by siNS5B, siNS4A and siNS5A, determined by western blotting. (**F**) Western blots for HCV core protein expression in Huh7.5.1 cells transfected with pCore-3FLAG (Huh7.5.1-Core cells). (**G**) immunofluorescent staining for HCV core protein expression in Huh7.5.1-Core cells. The cells were stained by the antibody against HCV core, and then FITC-labeled secondary antibody was applied (green fluorescence). The nuclei were counterstained with DAPI (blue). White scale bars = 200 μm. (**H**) Inhibition of HCV core gene expression in Huh7.5.1-Core cells by core-specific siRNAs (siRNA-core 1 and siRNA-core 2), determined by real-time PCR. ***p < 0.001, all siRNAs vs.Blank. (**I**) Inhibition of core protein expression in Huh7.5.1-Core cells by siRNA-core 1 and siRNA-core 2, determined by western blotting.

Following transfection with RNA transcribed from linearized pSGR-JFH1 and selection by cultivation with G418, the HCV-replicable cell line Huh7.5.1-HCV was constructed and the expression of HCV nonstructural protein 3 (NS3) and 5A (NS5A) were examined by western blotting and immunofluorescent staining assays. HCV NS3 and 5A were detected in Huh7.5.1-HCV, but not in Huh7.5.1 (Fig. 1A,B). Three siRNAs (siNS5B, siNS4A, siNS5A) were designed to target the replicon of HCV virus gene for destruction of its replication, their efficacy was examined by real-time PCR and western blot analysis for the expression of HCV NS3 or NS5A gene. All three siRNAs efficiently suppressed gene and protein expression of NS3 or NS5A (Fig. 1C–E), but the greatest efficacy was observed using siNS5A to target HCV replicon, so it was selected for subsequent experiments.

The plasmid pCore-3FLAG expressing the HCV core protein was transfected into Huh7.5.1 cells and core protein expression was examined at 48 h post-transfection by western blotting and immunofluorescent staining analysis (Fig. 1F,G). The core protein was detected using its specific antibody. Two core siRNA (siRNA-core 1 and siRNA-core 2) were designed to target the HCV core protein, and their efficacy was examined by real-time PCR and western blotting. Both core-specific siRNAs suppressed the core protein expression at the gene and protein levels (Fig. 1H,I), but the greatest efficacy was observed using siRNA-core 1, so it was selected for subsequent experiments.

### Characterization of nanoscale VE-DC/siRNA

**Figure 2:**
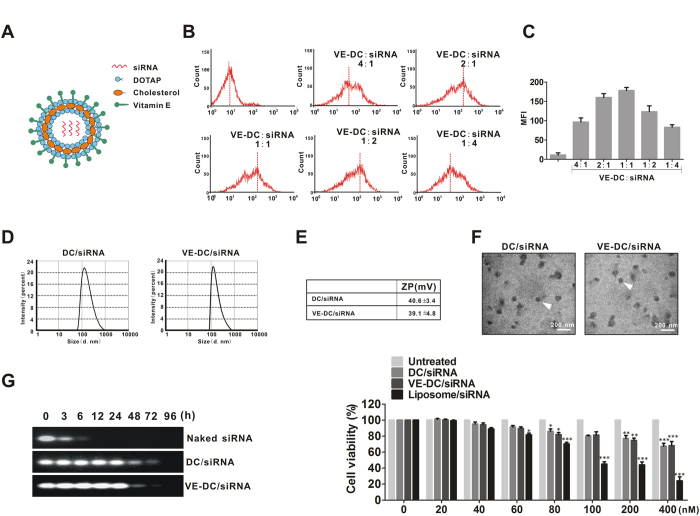
Characterization of nanoscale VE-DC/siRNA. (**A**) Schematic illustration of nanoscale VE-DC/siRNA. (**B**) Representative results for flow cytometer analysis of Huh7.5.1 cells transfected with fractionated molar ratios of VE-DC and siRNA-Cy3 ranging from 4:1 to 1:4. The experiment was repeated three times. (**C**) Statistical result for mean MFI ± SD of three independent experiments. (**D**) Representative result for the size distribution of VE-DC/siRNA and DC/siRNA. (**E**) Representative result for zeta potential of DC/siRNA and VE-DC/siRNA. ZP, zeta potential. (**F**) Transmission electron micrographs of DC/siRNA and VE-DC/siRNA. White scale bars = 200 nm. (**G**) Serum stability of nanoscale VE-DC/siRNA. (H) Cytotoxicities of various concentration of VE-DC/siRNA to Huh7.5.1-HCV cells. Results are expressed as the mean absorbances ± SD of three independent experiments. *p < 0.05, **p < 0.01 and ***p < 0.001, All vs.Untreated.

We determined the optimal VE-DC/siRNA ratio for transfection of siRNA (siNS5A) into Huh7.5.1 cells by incubating VE-DC carrying Cy3-labeled siRNA (VE-DC/siRNA-Cy3) in varied molar ratios ranging from 4:1 to 1:4. After transfection of Huh7.5.1 cells with fractionated molar ratios of VE-DC/siRNA-Cy3 (Fig. 2A), the transfection efficiency was determined using flow cytometry. The highest mean fluorescence intensity (MFI) was observed at a molar ratio of 1:1 in the incubation solution (Fig. 2B,C). We used complexes at this incubation molar ratio in subsequent experiments. The particle size distribution and zeta potential of the VE-DC/siRNA were determined at 37 °C with a laser particle size analyzer as 118.0 ± 7.7 nm and 40.6 ± 3.4 mV for DC/siRNA and 125.5 ± 9.0 nm and 39.1 ± 4.8 mV for VE-DC/siRNA (Fig. 2D,E). The VE-DC/siRNA had a spherical morphology and homogeneous distribution, as determined from TEM images (Fig. 2F), and its diameter was consistent with the results from the laser particle size analyzer.

Naked siRNA, DC/siRNA and VE-DC/siRNA in 50% human serum were compared for their *in vitro* stability. We observed intact siRNA even after 24 h in 50% serum in the DC/siRNA or VE-DC/siRNA groups. By contrast, naked siRNA was completely degraded within 6 h under the same conditions (Fig. 2G). These data suggested that the delivery vehicle may significantly improve the stability of siRNA in serum.

The cytotoxicity of VE-DC/siRNA was addressed by measuring cell viability by the CCK8 assay (Fig. 2H). The cell viabilities were much higher for the VE-DC/siRNA-treated Huh7.5.1 cells than for the cells treated with commercial liposomes. Treatment with a concentration of commercial liposomes of 100 nM resulted in only 45% viable cells. By contrast, DC/siRNA or VE-DC/siRNA treatments showed low cytotoxicity, even at concentrations up to 200 nM, which was much higher than the actual amount used for gene transfection.

### *In vitro* inhibition of HCV replication and core protein production by nanoscale VE-DC/siRNA

**Figure 3:**
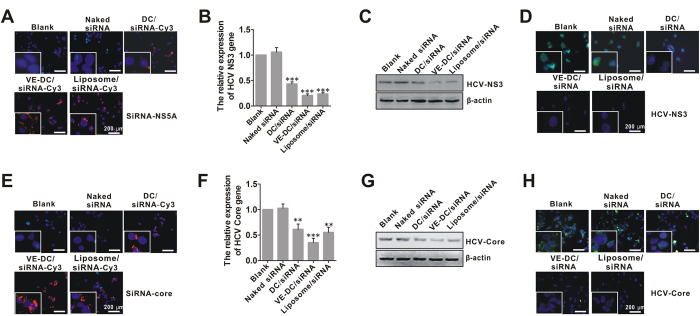
Inhibition of HCV replication in Huh7.5.1-HCV cells and core protein expression in Huh7.5.1-Core cells by VE-DC/siRNAs *in vitro*. (**A**) Representative fluorescent images of the intracellular distribution of VE-DC/siRNA-Cy3 in Huh7.5.1-HCV cells. The nucleus was stained with DAPI (blue). White scale bars = 200 μm. (**B**) Real-time PCR analysis for NS3 gene expression in naked siRNA-treated, DC/siRNA-treated, VE-DC/siRNA-treated, or liposome/siRNA (siRNA-NS5A)-treated Huh7.5.1-HCV cells for 24 h. ***p < 0.001, All vs.Blank. Liposome/siRNA was used as a positive control. (**C**) Western blot analysis for NS3 protein expression in naked siRNA-treated, DC/siRNA-treated, VE-DC/siRNA-treated, or liposome/siRNA-treated Huh7.5.1-HCV cells for 48 h. Liposome/siRNA was used as a positive control. (**D**) Immunofluorescent staining for NS3 protein expression in naked siRNA-treated, DC/siRNA-treated, VE-DC/siRNA-treated or liposome/siRNA-treated Huh7.5.1-HCV cells for 48 h. The cells were stained by the antibody against NS3, and then FITC-labeled secondary antibody was applied (green fluorescence). The nucleus was counterstained with DAPI (blue). White scale bars = 200 μm. Liposome/siRNA was used as a positive control. (**E**) Representative fluorescent images of the intracellular distribution of VE-DC/siRNA-Cy3 in Huh7.5.1-Core cells. (**F**) Real-time PCR analysis for core gene expression naked siRNA-treated, DC/siRNA-treated, VE-DC/siRNA-treated, or liposome/siRNA (siRNA-core)-treated Huh7.5.1-Core cells for 24 h. **p < 0.01 and ***p < 0.001, All vs.Blank. Liposome/siRNA was used as a positive control. (**G**) Western blot analysis for core protein expression in naked siRNA-treated, DC/siRNA-treated, VE-DC/siRNA-treated or liposome/siRNA-treated Huh7.5.1-Core cells for 48 h. Liposome/siRNA was used as a positive control. (**H**) Immunofluorescent staining analysis for core protein expression in naked siRNA-treated, DC/siRNA-treated, VE-DC/siRNA-treated, or liposome/siRNA-treated Huh7.5.1-Core cells for 48 h. The cells were stained with the antibody against the core protein, and then FITC-labeled secondary antibody was applied (green fluorescence). The nuclei were counterstained with DAPI (blue). White scale bars = 200 μm. Liposome/siRNA was used as a positive control.

We verified the uptake of VE-DC/siRNA by Huh7.5.1-HCV cells and elucidated its subcellular localization by monitoring VE-DC/siRNA-Cy3 (siNS5A)-treated Huh7.5.1-HCV cells using fluorescence microscopy (Fig. 3A). A denser granular pattern of fluorescence in the perinuclear region was seen for 24 h in VE-DC/siRNA-Cy3-treated Huh7.5.1-HCV cells than in the DC/siRNA-treated cells. Similar results were also confirmed from VE-DC/siRNA-Cy3 (siRNA-core)-treated Huh7.5.1-HCV Core cells (Fig. 3E).

We validated the suppressive effect of VE-DC/siRNA on HCV replication in Huh7.5.1-HCV cells by examining gene and protein expression of HCV NS3 by real-time PCR (Fig. 3B), western blotting (Fig. 3C), and immunofluorescent staining (Fig. 3D) assays. The gene and protein expression was significantly lower in VE-DC/siRNA-treated cells than in naked siRNA-treated or DC/siRNA-treated cells. The suppressive ability conferred by VE-DC/siRNA was further confirmed in VE-DC/siRNA (siRNA-core)-treated Huh7.5.1-Core cells (Fig. 3F–H).

### Silencing of target genes in liver of mice by VE-DC/siRNA

**Figure 4:**
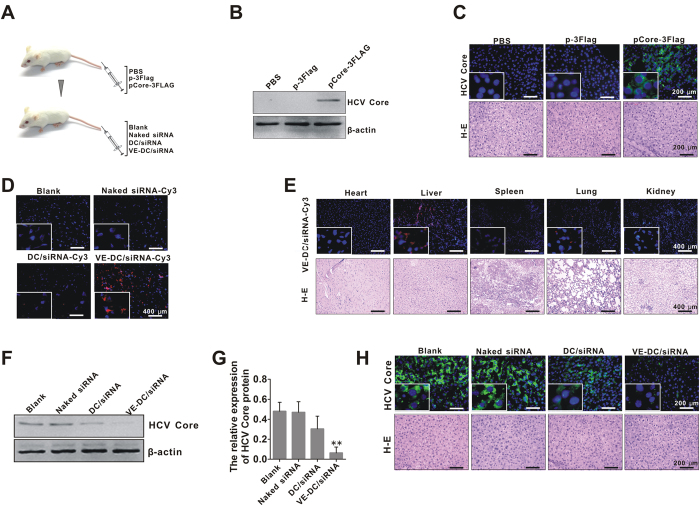
Silencing of the target HCV core gene in the mouse liver by VE-DC/siRNA. (**A**) Schematic representation of the experimental procedure in mice. (**B**) Western bolt analysis for representative liver-specific expression of core in mice hydrodynamically injected with the pCore-3FLAG (5 μg/mouse) for 48 h. β-actin was used as an input control. (**C**) Representative immunofluorescent staining result for core protein expression in mice hydrodynamically injected with the pCore-3FLAG 48 h. Sections of liver tissue were stained with the antibody against the core protein, and then FITC-labeled secondary antibody was applied (green fluorescence). The nuclei were counterstained with DAPI (blue). White scale bars = 200 μm. (**D**) Representative fluorescent images of the liver distribution of Cy3 signal in mice at 1 h after injection of naked siRNA-Cy3, DC/siRNA-Cy3 and VE-DC/siRNA-Cy3. The nuclei were stained with DAPI (blue). White scale bars = 400 μm. (**E**) Representative fluorescent images of Cy3 signal in heart, liver, spleen, lung, and kidney at 1 h after injection of VE-DC/siRNA-Cy3. The nuclei were counterstained with DAPI (blue). White scale bars = 400 μm. (**F**) Representative western blot result for liver-specific expression of core in mice hydrodynamically injected with the pCore-3FLAG (5 μg/mouse) and then treated intravenously, 24 h later, with and without naked siRNA, DC/siRNA and VE-DC/siRNA at an siRNA dose of 0.8 mg/kg per mouse for 24 h. (**G**) The relative expression of the core protein is quantified by core/β-actin densitometric ratio in mice (n = 5/each group) treatment as mentioned in F. **p < 0.01, VE-DC/siRNA vs.Blank. (**H**) Representative immunofluorescent staining result for liver-specific expression of the core protein in mice treatment as mentioned in F. Sections of liver tissue were stained with the antibody against the core protein, and then FITC-labeled secondary antibody was applied (green fluorescence). The nuclei were counterstained with DAPI (blue). White scale bars = 200 μm.

The liver-specific expression of the HCV core gene was established by hydrodynamic tail vein injection of plasmid pCore-3FLAG in Balb/c mice (Fig. 4A). The core protein expression was evaluated by western blotting (Fig. 4B) and immunofluorescent staining (Fig. 4C) assays. We assessed the *in vivo* silencing ability of VE-DC/siRNA (siRNA-core) by administering VE-DC/siRNA to this mouse model, which harbors liver-specific expression of the core protein. We first investigated whether delivery of VE-DC/siRNA-Cy3 to the liver had been successful. The mice were injected with VE-DC/siRNA-Cy3 via the tail vein and the livers were extirpated and observed by fluorescence microscopy at 1 h after injection. We observed a marked accumulation of the Cy3 signal in the liver, while a weak signal was found in the control liver sections from the mice injected with naked siRNA-Cy3 or DC/siRNA-Cy3 (Fig. 4D). These results demonstrated that VE-DC was more effective at delivering siRNA into the liver cells.

We also investigated the *in vivo* distribution of siRNA delivered by VE-DC. At 1 h after injection, the fluorescence intensity was higher in the liver than in other internal organs, such as heart, spleen, lung, and kidney, which suggested that VE-DC facilitated the liver-specific cellular uptake of siRNA (Fig. 4E). Mice were also sacrificed on the second day after injection of VE-DC/siRNA for measurement of expression of core protein in the liver by western blotting (Fig. 4F,G) and immunofluorescent staining (Fig. 4H) assays. The DC/siRNA inhibited core protein expression by up to 36.17% in the livers of some mice (probability, one in five), but this non-targeted delivery was not statistically significant. By contrast, VE-DC/siRNA reduced the target core protein by an average of 83.01%. Taken together, these results suggest that VE-DC facilitated liver-specific cellular uptake of siRNA, which subsequently suppressed HCV core protein production.

**Figure 5:**
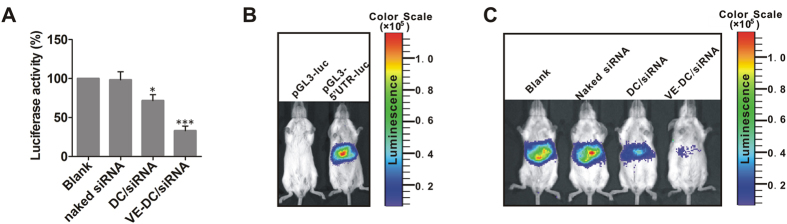
Inhibition of luciferase gene expression by VE-DC/siRNA both *in vitro* and *in vivo*. (**A**) Repressive effect of VE-DC/siRNA (siRNA-5′UTR) on relative luciferase activities in Huh7.5.1 cells co-transfected with pRL-PK and pGL3-5′UTR-luc. The results are expressed as mean ± SD of 3 independent experiments. The relative luciferase activity of that transfected with pGL3-5′UTR-luc in control group was set as 1. *p < 0.05, ***p < 0.001, all vs.Blank. (**B**) Representative whole body images for luciferase expression in BALB/c mice 48 h after hydrodynamic injection with the pGL3-5′UTR-luc. (**C**) Representative whole body images for luciferase expression in BALB/c mice hydrodynamically injected with the luciferase expression plasmid pGL3-5′UTR-luc and then 24 h later treated intravenously with and without naked siRNA, DC/siRNA or VE/DC-siRNA (siRNA-5′UTR) at an siRNA dose of 0.8 mg/kg per mouse (n = 3/each).

We validated the repressive effect of VE-DC/siRNA on HCV replication using DC/siRNA (siRNA-5′UTR) or VE-DC/siRNA (siRNA-5′UTR) to treat the Huh7.5.1 cells. The cells were co-transfected with pRL-PK Renal luciferase and pGL3-5′UTR-luc, and the relative luciferase activity was detected by a multiple function enzyme analyzer. The relative luciferase activity was significantly lower in VE-DC/siRNA-treated cells than in naked siRNA-treated or DC/siRNA-treated cells (Fig. 5A). We evaluated the destructive effect of VE-DC/siRNA on HCV replication in a model of liver-specific expression of luciferase regulated by HCV 5′UTR, which we established by the hydrodynamic tail vein injection of plasmid pGL3-5′UTR-luc in Balb/c mice (Fig. 5B). These mice transiently expressed luciferase in the liver, and we determined the changes in liver-specific expression of luciferase by whole body image analysis 24 h after injection of VE-DC/siRNA (siRNA-5′UTR) (Fig. 5C). The relative luciferase activity was significantly lower in the VE-DC/siRNA group than in the naked siRNA-treated or DC/siRNA-treated cells, suggesting that VE-DC facilitated liver-specific cellular uptake of siRNA and subsequent efficient silencing of HCV replication.

### No side effects are produced by VE-DC/siRNA

**Figure 6:**
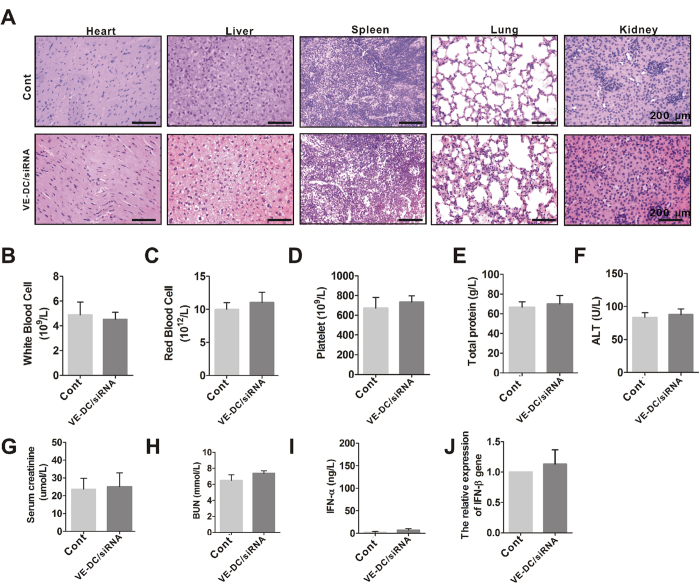
*In vivo* side effects of the VE-DC/siRNA. (**A**) Representative histopathology for different organs in mice treated with and without VE-DC/siRNA for 24 h by hematoxylin-eosin staining (H-E). Blank scale bars = 200 μm. (**B**) Blood samples were drawn from orbit in mice treated with and without VE-DC/siRNA for 24 h and WBC levels were assessed. Values shown are means ± SD (n = 8/each group). (**C**) RBC from mice treated with and without VE-DC/siRNA was assessed Values shown are means ± SD (n = 8/each group). (**D**) Platelets from mice treated with and without VE-DC/siRNA were assessed. Values shown are means ± SD (n = 8/each group). (**E**) Total protein levels from mice treated with and without VE-DC/siRNA were assessed. Values shown are means ± SD (n = 8/each group). (**F**) ALT levels from mice treated with and without VE-DC/siRNA were assessed. Values shown are means ± SD (n = 8). (**G**) Creatinine levels from mice treated with and without VE-DC/siRNA were assessed. Values shown are means ± SD (n = 8/each group). (**H**) BUN levels from mice treated with and without VE-DC/siRNA were assessed. Values shown are means ± SD (n = 8/each group). (**I**) ELISA analysis for serum IFN-a level derived from mice treated with and without VE-DC/siRNA for 3 h. Values shown are means ± SD (n = 5/each group). (**J**) Real-time PCR analysis of IFN-β gene expression from mice (n = 5/each group) treated with and without VE-DC/siRNA for 3 h.

In addition to therapeutic efficacy, we also evaluated the *in vivo* side effects of the VE-DC/siRNA. Hematoxylin-eosin (H-E) staining results indicated no noticeable histological changes in the tissues from heart, liver, spleen, lung, and kidney, suggesting no organ toxicity (Fig. 6A). The complete blood count (CBC) results showed that white blood cells, red blood cells, and platelets did not differ between the control group and VE-DC/siRNA group (Fig. 6B–D). In addition, no abnormalities were evident in biochemical parameters, including total protein, alanine aminotransferase (ALT), blood urine nitrogen (BUN), and creatinine (Fig. 6E–H). These results suggest that VE-DC/siRNA had no effect on the blood system, on liver and kidney functions, and on the histopathology of organs.

We examined whether VE-DC/siRNA caused a type I IFN response by measuring the induction of interferons (IFNs) in serum 3 h after the injection of VE-DC/siRNA. The serum level of IFN-α was barely detectable by ELISA (Fig. 6I), and no increases in IFN-β gene expression were observed in the liver by real-time PCR (Fig. 6J). These results suggest that VE-DC/siRNA does not trigger an immune reaction.

## Discussion

Safe and efficient gene delivery is a major task that requires the development of new gene vectors with low cytotoxicity and high gene transfection performance. Previous studies have used hydrodynamic injection^27^, viral vectors^28^, and cationic liposomes^29^ as the main methods of *in vivo* gene delivery. However, hydrodynamic injection is unsuitable for human therapy, and application of viral vectors may result in severe side effects. By contrast, since the physical properties of cationic liposomes are well understood, liposomes are good prospects for clinical applications. In this paper, we designed and synthesized a novel siRNA-packing nanoscale cholesterol-based cationic liposomes aimed at destroying HCV RNA and coupled this with VE promoted delivery of siRNA to the liver. The biological behaviors of the VE-DC/siRNA platform were evaluated and confirmed high delivery efficiency and low toxicity, indicating it as potential gene delivery vector.

Several delivery systems for siRNA based on liposomes show great promise due to their favorable characteristics, such as the ease of large-scale production and biocompatibility. Their use in gene therapy is currently under investigation in several clinical trials for the treatment of diseases^30^. However, the use of liposomes as siRNA carriers has some limitations in terms of gene transfection efficiency, the need for high dose injections, and poor targeted tissue distribution *in vivo*^31^. An *in vivo* study in mice showed that intravenous injection of DOTAP/siRNA complexes had an effective effect than injection of naked siRNA, but it also produced potent cytotoxicity and immune responses^32^. Conjugation of cholesterol to siRNA molecules could improve the delivery efficiency of siRNA targeting apolipoprotein B in the liver, but it required high doses of cholesterol-conjugated siRNA (50 mg/kg), which limits their therapeutic applications in humans due to cost^33^. Cholesterol-conjugated siRNA has also been reported to silence the herpes simplex virus-related genes after intravaginal administration without inflammation or interferon response at the administrative site, but it still need a high dose of cholesterol/siRNA^34^. The gene transfection efficiency and cytotoxicity strongly depend on the molecular backbone of the delivery vectors. Thus, the selection of appropriate molecular building blocks and construction of low cytotoxicity and highly efficient gene vectors are important in gene therapy.

Vitamins are organic compounds and essential for liver tissue cells, but they cannot be synthesized within the cells. Recent studies have demonstrated that modification of delivery systems with vitamins is a very promising strategy for liver targeting^35^,^36^,^37^. Vitamin E is an antioxidant, and its uptake strengthens the immune system response against viruses and bacteria, and its uptake is not toxic, even at high doses^38^,^39^,^40^. The natural isomer of VE, α-tocopherol, is abundant in human diets and easily absorbed^41^. For these reasons, we designed (VE)-coupled lipid nanoparticles carrying siRNAs against the HCV replicon, the 5′UTR, and the core gene.

Hydrodynamic gene delivery using a rapid injection of a relatively large volume of DNA solution has opened up a new avenue for gene studies *in vivo*. This method is simple and effective. Wide success in applying hydrodynamic principles to delivery of DNA, RNA, proteins, and synthetic compounds, into the cells in various tissues of small animals, has inspired the recent attempts at establishing a hydrodynamic procedure for clinical use^42^,^43^. In our present study, the liver-specific expression of the HCV core or liver-specific expression of luciferase regulated by HCV 5′UTR was established by hydrodynamic tail vein injection of plasmid pCore-3FLAG or pGL3-5′UTR-luc in Balb/c mice. Nanoscale VE-DC/siRNA (siRNA-core or siRNA-5′UTR) was intravenously injected to assess the liver-targeting property as well as the antiviral activity. The importance of VE in the delivery of siRNAs *in vivo* was examined by comparing the *in vivo* silencing activity of VE-coupled lipid nanoparticles (VE-DC) with equal amounts of uncoupled DC. The VE-coupled DC enabled the efficient *in vivo* delivery of siRNA to the liver, and resulted in a more efficient silencing effect. The relatively low dose of siRNA (0.8 mg/kg per single injection) used in our *in vivo* study was less than doses previously shown to have *in vivo* therapeutic effects^44^,^45^. Therefore, coupling of DC with VE not only increases the liver targeting property but also facilitates the liver-specific cellular uptake of siRNA, which efficiently suppressed HCV replication and HCV core protein production.

Previous studies have suggested that LDL particles can mediate the delivery of cholesterol-siRNAs into liver tissues and that the particles then enter hepatocytes through a lipoprotein receptor-mediated pathway^46^. Our data showed an enhanced liver-targeting property and liver-specific cellular uptake after coupling of lipid nanoparticles with VE. In contrast to cholesterol, VE is an exogenous lipid that cannot be synthesized *in vivo*, suggesting that its transport mechanism and cellular uptake in the liver might differ from those of cholesterol. One possible mechanism might involve a high binding affinity of VE to the novel binding glycoprotein, afamin, which shows multiple VE-binding sites and is highly expressed in the liver^47^,^48^. The possible role of afamin in facilitating liver-specific cellular uptake of VE-DC/siRNAs will be elucidated in future studies. The intracellular fate of VE-DC/siRNAs might involve degradation of the VE-DC/siRNAs in endolysosomes, release of siRNAs into the cytosol, and binding to target mRNA.

Notably, no prominent *in vivo* side effects were noted after intravenous injection of VE-DC/siRNAs in our blood system, biochemical, and histopathology analyses. The dosage of α-tocopherol, when used as a nutritional supplement is 15 mg/day^49^. When 0.8 mg/kg of VE-DC/siRNA was injected, the amount of α-tocopherol delivered was only 6.5 μg for each mouse, which is a very small dose. The formulation in lipid nanoparticles can activate the innate immune responses through activation of Toll-like receptors, which has direct effects in modulating viral replication^50^,^51^. We excluded the possibility that the anti-HCV effect of VE-DC/siRNAs might be confounded by the immune stimulation of an innate immune response by investigating the IFN-α/β response, which involves the expression of the IFN-β and IFN-α genes. Of specific interest is the finding that VE-DC/siRNA did not induce an increase of IFN-α in the serum or of IFN-β mRNA in the liver. One possible mechanism of escape from an immunostimulatory effect in VE-DC/siRNA-injected mice could reflect the use of low doses of cholesterol-based cationic liposome DC in our VE-DC, which might not have been sufficient to induce an immunostimulatory effect. The other possible mechanism is that composition of nanoscale VE-DC with VE may formulate a different molecular backbone which is different from before, VE itself might exert an unclear neutralize effect on *in vivo* side effects. So these possibilities should be verified in our further research. The results presented here, taken together, indicate that VE-DC/siRNAs are a noninvasive delivery vector for siRNA.

In summary, we confirmed the effectiveness and safety of VE-coupled siRNA-packing nanoscale cholesterol-based cationic liposomes for *in vivo* delivery of siRNA. The actual delivery pathway of VE-DC-siRNA remains to be established for better optimization of its use, but this study represents an important step in advancing the use of gene delivery vectors potentially very useful systems for gene therapy.

## Methods

### Plasmid DNA

The plasmid pSGR-JFH1 (DDBJ/EMBL/GenBank accession number AB114136) containing a HCV 2a subgenomic replicon (JFH1) (provided by Takaji Wakita, Tokyo Metropolitan Institute for Neuroscience, Tokyo, Japan) was used to construct a HCV-replicable cell line, Huh7.5.1-HCV. The plasmid pCDNA3.1(+)-3FLAG-Core (pCore-3FLAG) expressing FLAG-tagged core protein was constructed by PCR amplification of the core from plasmid HFL (provided by Dr. Rice, Rockefeller University, USA) containing a full length of HCV 1a and subcloned into the eukaryotic expression vector pCDNA3.1(+)-3FLAG (p-3FLAG). The plasmid containing the full sequence of the HCV genome 5′UTR (pGL3-5′UTR-luc) was constructed by PCR amplification from plasmid HFL and subcloned into the Luciferase Reporter Vector, pGL3-luciferase (pGL3-luc). The HCV genome 5′UTR was used as the promoter for reporter gene luciferase. All these plasmids were constructed previously in our laboratory and are shown in Figure. S1.

### Reagents and antibodies

The primary antibodies used for this study were as follows: the mouse anti-HCV NS5A monoclonal antibody [H26] (Abcam, USA), mouse anti-HCV NS3 monoclonal antibody (Abcam, USA), goat anti-HCV core polyclonal antibody (Santa Cruz, USA), and mouse anti-β-actin monoclonal antibody (Boster, China). DAPI (Santa Cruz, USA) and Lipofectamine 2000 (Invitrogen, USA) was used per the manufacturer’s instructions.

### Cell culture

The human hepatocellular carcinoma cell line Huh7.5.1 was maintained in the Dulbecco’s modified Eagle’s medium (DMEM) supplemented with 10% fetal bovine serum (FBS; Gibco, USA), 100 U/ml penicillin, and 100 μg/ml streptomycin. The replication ability of the JFH-1 replicon in Huh7.5.1 cells was established by transfecting RNA transcribed from linearized pSGR-JFH1 into Huh7.5.1 cells. Transfected cells were cultured for 3 weeks with G418 (Sigma, USA), resulting in an HCV-replicable cell line (Huh7.5.1-HCV). All other procedures were performed as previously described^52^. The Huh7.5.1-HCV cells were maintained in DMEM supplemented with 10% FBS, 100 U/ml penicillin, 100 μg/ml streptomycin and 400 μg/ml G418. The cell culture was maintained at 37 °C in a humid atmosphere containing 5% CO_2_.

### Synthesis of siRNAs

The siRNAs against the HCV 2a replicon (JFH1), HCV 1a 5′UTR, and the core gene were designed to the most conserved target region of these genes using the Ambion’s siRNA desigion tool http://www.ambion.com/techlib/misc/siRNA_finder.html. Negative control siRNAs (scrambled siRNAs) were designed using scrambled sequences. All siRNAs were chemically synthesized by invitrogen (USA) and are shown in Table S1.

### Preparation and characterization of nanoscale VE-DC/siRNA

Cholesterol-based cationic liposomes were prepared by the thin-film hydration method^53^. Briefly, 1,2-dioleoyl-3-trimethylammoniumpropane (DOTAP) (Avanti, USA) and cholesterol (GENVIEW, USA) (molar ratio = 1:1) were dissolved in 10 ml trichloromethane. Removal of the organic solvent by rotary evaporation under vacuum at 40 °C, resulted in a thin film, which was then combined with 10 ml trichloromethane and 3 ml phosphate buffered saline (PBS) and sonicated with a probe sonicator at 100 W for 2 min at 37 °C to form a mixture. The mixture was rotated under a partial vacuum at 40 °C to prepare a gel state, and then 7 ml PBS was added. The gel state was rotated for 30 min at ordinary pressure at 40 °C, resulting in the formation of a cationic liposome suspension, referred to as DC. Vitamin E coupled DCs (VE-DCs) were prepared by mixing vitamin E (SIGMA, USA) with the cationic liposome suspensions at a 1:2 (mol/mol) ratio overnight at 4 °C. The VE-coupled DCs carrying siRNA (VE-DC-siRNA) were prepared by mixing a solution of siRNA with VE-coupled liposomes at a 1:1 (mol ∕mol) ratio for 10 min at 37 °C. The size and surface charge (zeta potential) of the nanoscale VE-DC/siRNA were determined with a laser particle size analyzer (Rise-2008, China) and the experiment was repeated three times. The homogeneity and dispersivity of the samples were analyzed by transmission electron microscopy (HITACHI7500, Japan). The optimal molar ratio of VE-DC and siRNA for transfection of siRNA into Huh7.5.1 cells was determined by treating the cells with VE-DC carrying Cy3-labeled siRNA (Cy3 bound to the sense strand of siRNA) (VE-DC/siRNA-Cy3) in fractionated molar ratios ranging from 4:1 to 1:4 for 24 h. The transfection efficiency of Huh7.5.1 cells by VE-DC/siRNA with fractionated molar ratios was then determined by flow cytometry (Becton Dickinson, USA).

### *In vitro* stability assays

Naked siRNA, DC/siRNA, and VE-DC/siRNA were incubated in 50% human serum at 37 °C for 0, 3, 6, 12, 24, 48, 72, and 96 h. Aliquots taken at different time points were treated with 0.1% SDS and immediately stored at 72 °C. All samples were separated in 2% agarose gels and stained with Good View^TM^ (SBS Genetech, China). The results were recorded with a gel imaging system (Gel Doc 1000, Bio-Rad, USA).

### *In vitro* cytotoxicity of VE-DC/siRNA

The CCK-8 assay was used to assess the cytotoxicity of VE-DC/siRNA using a Cell Counting Kit (Dojindo, Japan) following the manufacturer’s protocol. The data were reported as the fold change over the untreated control.

### Cellular uptake of VE-DC/siRNA

Huh7.5.1 and Huh7.5.1-HCV cells were plated onto cleaned-up cover slips. DC/siRNAs-Cy3, VE-DC/siRNAs-Cy3, and liposome/siRNAs-Cy3 were added to the cells at a final siRNA concentration of 40 nM. At 24 h post-treatment, the cells were washed with PBS, fixed with 4% paraformaldehyde, and exposed to DAPI for 1 min to stain nuclei. The subcellular localization of Cy3-labeled siRNAs was assessed by fluorescence microscopy (Leica DM4000, Germany).

### Immunofluorescent staining

Cells or frozen sections were washed with PBS and fixed in 4% paraformaldehyde, then permeabilized with 0.2% Triton X-100. Cover slips were rinsed and incubated with blocking serum and then incubated overnight at 4 °C with primary anti-HCV Core or anti-HCV NS3 antibody. The cells were then washed with PBS and stained with the corresponding FITC-conjugated secondary antibody. The nuclei were visualized by staining the cells with DAPI. The fluorescent images were then observed and analyzed by fluorescence microscopy.

### Real time quantitative PCR analysis

Cells were transfected with DC/siRNAs, VE-DC/siRNAs, or liposome/siRNAs for 24 h and then lysed with Trizol (Invitrogen, Carlsbad, CA, USA). Complementary single-stranded DNA was synthesized from total RNA by reverse transcription (TaKaRa, Japan). Quantification of cDNA targets was performed on CFX96TM Real-Time-PCR Detection System (Bio-Rad, USA). Primers were synthesized by Invitrogen and are listed in Table S2.

### Western blotting

The levels of HCV NS3, NS5A, and core protein in cells or liver tissues were evaluated by western blotting. Briefly, samples containing equal amount of protein were separated by SDS-PAGE and blotted onto PVDF membranes. The membranes were blocked with 5% bovine serum albumin and incubated with anti-HCV Core, anti-HCV NS3, anti-NS5A, or anti-β-actin antibodies, following by incubation with secondary antibodies conjugated with horseradish peroxidase. The proteins of interest were detected using the SuperSignal West Pico Chemiluminescent Substrate kit (ThermoFisher Scientific, USA). The results were recorded by the Bio-Rad Electrophoresis Documentation (Gel Doc 1000, Bio-Rad, USA) and Quantity One Version 4.5.0.

### *In vitro* luciferase assay

The luciferase assay was performed as previously described^54^. Briefly, the Huh7.5.1 cells were seeded and grown in each well of 24-well culture plates, transfected with plasmid transfection mix (pRL-PK Renal luciferase, 100 ng; pGL3-5′UTR-luc or pGL3-luc, 900 ng) for 24 h, and then treated with DC/siRNA or VE-DC/siRNA for another 24 h. The cells were then lysed in 1× luciferase lysis buffer (Promega, USA), and the lysates were assayed for luciferase activity on a multiple function enzyme analyzer. The firefly luciferase activity was normalized for the transfection efficiency based on renilla luciferase activity.

### Animals

Female BALB/c mice, 6 weeks of age, were purchased from the animal center at Chongqing Medical University. All the *in vivo* experiments were approved and conducted in accordance with the guidelines established by the University Animal Care and Use Committee for Laboratory Animal Research in Chongqing Medical University.

### *In vivo* distribution of siRNA delivered by VE-DC

VE-DC/siRNA-Cy3 was intravenously injected into BALB/c mice. At 1 h after injection, the livers and other organs (heart, lungs, spleen, and kidneys) were harvested from each mouse. Tissue specimens were immediately embedded in OCT medium and cryogenically sectioned. Sections of these tissues were then stained with DAPI, and slides were finally examined by fluorescence microscopy.

### *In vivo* HCV siRNA treatment

We evaluated the effect of siRNA treatment on HCV by conducting two sets of experiments. The first set of experiments involved the constructions of an HCV mouse model by hydrodynamic injection of 5 μg pCore-3FLAG or control p-3FLAG. Twenty-four hours later, PBS, naked siRNA, DC/siRNA (siRNA-core, 0.8 mg/kg), or VE-DC/siRNA (siRNA-core, 0.8 mg/kg) was intravenously administered (n = 4, each group). On day 2 after siRNA treatment, the mice were sacrificed and liver tissues were homogenized. The HCV core protein level was determined by immunofluorescent staining and western blotting. The second set of experiments involved the construction of the other HCV mouse model by hydrodynamic injection of 5 μg pGL3-5′UTR-luc. 24 h later, PBS, naked siRNA (siRNA-5′UTR, 0.8 mg/kg), DC/siRNA (siRNA-5′UTR, 0.8 mg/kg) or VE-DC/siRNA (siRNA-5′UTR, 0.8 mg/kg) were intravenously administered. On day 2 after siRNA treatment, whole body images showing RNAi activity were analyzed using a Xenogen IVIS Spectrum instrument (PERKIN ELMER, USA).

### Hematological and serum biochemical and histological analysis

Blood samples were drawn from the orbits of mice 24 h after injection of VE-DC/siRNA. Hematological parameters (white blood cells, red blood cells, and platelets) were determined by a full automatic blood cell analysis (SYSMEX XS-500i, Japan). The total protein, alanine aminotransferase (ALT), blood urine nitrogen (BUN), and creatinine levels in serum were assayed using an automated chemical analyzer Hitachi7600-110 (Japan). Serum IFN-a level was detected using an ELISA Kit (DGE, China) following the manufacturer’s protocol. Heart, liver, spleen, lung, and kidney tissues were collected from mice 24 h after injection of VE-DC/siRNA, fixed and embedded in paraffin wax, and then sectioned into 5 μm thick slices. Then sections were stained with H-E and examined by light microscopy for alteration of histological structures.

### Statistical analysis

All values in the text and figures are presented as the means ± standard deviation (SD). The differences were analyzed using t test and one-way ANOVA followed by the Student-Newman-Keuls test, and all statistical analyses were performed using GraphPad Prism software (GraphPad Software, La Jolla, California, USA). Statistical differences are presented at probability levels of *p < 0.05, **p < 0.01 and ***p < 0.001.

## Additional Information

**How to cite this article**: Duan, L. *et al*. Target delivery of small interfering RNAs with vitamin E-coupled nanoparticles for treating hepatitis C. *Sci. Rep*. **6**, 24867; doi: 10.1038/srep24867 (2016).

## Supplementary Material

Supplementary Information
