# Supplementary material for: Target delivery of small interfering RNAs with vitamin E-coupled nanoparticles for treating hepatitis C
Source: Sci Rep. 2016 Apr 26;6:24867. doi: 10.1038/srep24867 (PMC4845054; doi:10.1038/srep24867)
Supplement: Supplementary Information [file srep24867-s1.doc]

Target delivery of small interfering RNAs with vitamin E-coupled nanoparticles for treating hepatitis C

Liang Duan*, Yan Yan*, Jingyi Liu, Bo Wang, Pu Li, Qin Hu & Weixian Chen

**Figure S1**

**
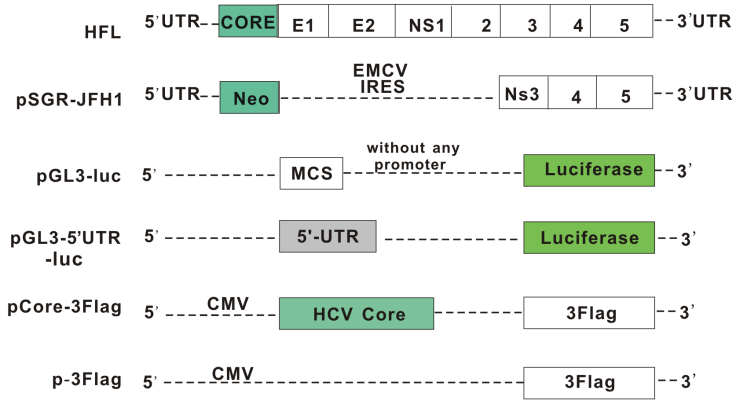
**

**Fig. S1. Schematic representation of plasmids used in our study**. UTR, untranslated region; NS, nonstructural protein 3; EMCV IRES, encephalomyocarditis virus internal ribosome entry site; CMV, cytomegalovirus promoter.

Table S1. Sequences of target siRNAs

| **siRNAs used for silencing** | |
| --- | --- |
| SiNS5B | S:GACCAAGCUCAAACUCACUdTdT |
|  | A:AGUGAGUUUGAGCUUGGUCdTdT |
| SiNS4A | S: UGGCCCAAAGUGGAACAAUdTdT |
|  | A:AUUGUUCCACUUUGGGCCAdTdT |
| SiNS5A | S: UCAAGCUAGCGGCUUCCAAdTdT |
|  | A: UUGGAAGCCGCUAGCUUGAdTdT |
| Si-Core-1 | S: GGUUGGUGUUACGUUUGGUdTdT |
|  | A: ACCAAACGUAACACCAACCdTdT |
| Si-Core-2 | S: CGUAAGGGUAUCGAUGACCdTdT |
|  | A:GGUCAUCGAUACCCUUACGdTdT |
| SiRNA-5’UTR | S: GCGUUAGUAUGAGUGUCGUdTdT |
|  | A:ACGAGACUCAUACUAACGCdTdT |
| Scramble siRNA | S: GUCGAGUCGCGUAUGCAGGdTdT |
|  | A:CCUGCAUACGCGACUCGACdTdT |

S: sense; A: antisense.

Table S2. Sequences of primers

| **Primers used in Real-Time PCR** | |
| --- | --- |
| Core 1a | S:GGACGACGATGACAAGGACT |
| Core 1a | A: GGGGAGACAGGA GCCATC |
| NS3 | S: CTGGGACCAAGTCTTTGGAG |
| NS3 | A: CCTTCAAGGTCGAAATGGGT |
| NS5A | S: AACTACAAGACCGCCATCTG |
| NS5A | A: GGTGCAAACCTATGGATCTG |
| hGAPDH | S:TTCTCTGATTTGGTCGTATTGG |
| hGAPDH | A:CATGTAAACCATGTAGTTGAGG |
| mIFN-β | S: AAGAGTTACACTGCCTTTGCCATC |
| mIFN-β | A:CACTGTCTGCTGGTGGAGTTCATC |
| mGAPDH | S: GGCTGCCCAGAACATCAT |
| mGAPDH | A:CGGACACATTGGGGGTAG |

**S:** sense strand; A: anti-sense strand.
